# Increasing Accumulation of Perfluorocarboxylate Contaminants Revealed in an Antarctic Firn Core (1958–2017)

**DOI:** 10.1021/acs.est.2c02592

**Published:** 2022-07-26

**Authors:** Jack Garnett, Crispin Halsall, Holly Winton, Hanna Joerss, Robert Mulvaney, Ralf Ebinghaus, Markus Frey, Anna Jones, Amber Leeson, Peter Wynn

**Affiliations:** † Lancaster Environment Centre, Lancaster University, Lancaster LA1 4YQ, U.K.; ‡ British Antarctic Survey, Cambridge, High Cross, Madingley Road, Cambridge CB3 0ET, U.K.; § Antarctic Research Centre, Victoria University of Wellington, Wellington 6012, New Zealand; ∥ Helmholtz-Zentrum Hereon, Max-Planck-Straße 1, 21502 Geesthacht, Germany

**Keywords:** PFAS, Antarctica, industrial emissions, CFCs, global regulation

## Abstract

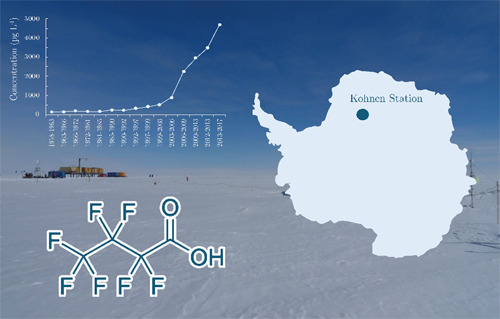

Perfluoroalkyl acids (PFAAs) are synthetic chemicals with a variety of industrial and consumer applications that are now widely distributed in the global environment. Here, we report the measurement of six perfluorocarboxylates (PFCA, C_4_–C_9_) in a firn (granular compressed snow) core collected from a non-coastal, high-altitude site in Dronning Maud Land in Eastern Antarctica. Snow accumulation of the extracted core dated from 1958 to 2017, a period coinciding with the advent, use, and geographical shift in the global industrial production of poly/perfluoroalkylated substances, including PFAA. We observed increasing PFCA accumulation in snow over this time period, with chemical fluxes peaking in 2009–2013 for perfluorooctanoate (PFOA, C_8_) and nonanoate (PFNA, C_9_) with little evidence of a decline in these chemicals despite supposed recent global curtailments in their production. In contrast, the levels of perfluorobutanoate (PFBA, C_4_) increased markedly since 2000, with the highest fluxes in the uppermost snow layers. These findings are consistent with those previously made in the Arctic and can be attributed to chlorofluorocarbon replacements (e.g., hydrofluoroethers) as an inadvertent consequence of global regulation.

## Abstract 2

Perfluorocarboxylic acids show increasing accumulation in a remote Antarctic firn core despite international regulation on their production.

## 1 Introduction

Poly- and per-fluorinated alkylated substances (PFAS) are a large group of synthetic chemicals that have been manufactured since the 1950s. Their chemical structure often combines a fully fluorinated hydrophobic carbon chain of varying lengths (typically C_3_–C_15_), with a hydrophillic terminal functional group (e.g., COO^–^, SO_3_^–^ etc.), providing surfactant properties. As such, PFAS have been used extensively in a wide variety of consumer products such as stain and water repellents, as processing aids in the manufacture of fluoropolymers and as surfactants in firefighting foams. They are also highly resistant to degradation and persist in the environment.^1^

Perfluoroalkyl acids (PFAA) are a key group of PFAS that consist of perfluorocarboxylates (PFCA) and perfluoroalkane sulfonates (PFSA). In 2000, the largest fluorochemical producer, 3 M Co., began phasing out the production of one major PFAA, perfluorooctane sulfonic acid (PFOS), over concerns of its harmful impacts on the environment, including its ability to bioaccumulate and elicit a series of adverse health effects in humans and wildlife.^2^ PFAA are discharged directly into the environment from industrial sources^3^ but are also formed indirectly through the photooxidation of volatile precursor compounds in the atmosphere, which are also released as industrial emissions.^4−11^ As a result, PFAA are considered global contaminants and a growing number, including PFAA-precursor compounds are now regulated and listed in Annexes of the UNEP’s Stockholm Convention on Persistent Organic Pollutants.

To meet ongoing and increasing global demand for high-performance surfactants, one approach by industry has been to replace more bioaccumulative long-chain PFAA with short-chain analogues.^12^ However, while many of these substitutes show comparable industrial functions, they are similarly persistent, more mobile in the environment,^13^ and their somewhat inferior technical performance is often compensated by higher usages.^14^ A second outcome of chemical regulation has led global PFAS production to shift regionally from Western countries (e.g., United States) to locations where regulation is less stringent (e.g., China), thus having the potential of offsetting the global health benefits of regulation of these chemicals. Finally, other global regulations such as the Montreal Protocol and the Kyoto Protocol have driven the development of new chemicals, which display low ozone-depleting potential (ODP) and low global warming potential (GWP), respectively. However, these substances are used in vast quantities (e.g., chlorofluorocarbon replacements) as refrigerants across the globe and have been revealed to breakdown in the environment to also form PFAA.^6,15^ Clearly, an effective global monitoring strategy is needed to assess the global impact of regulations, shifting geographical locations and changing chemical sources.^16^

The mechanisms by which PFAS are transported over long distances to remote Polar, marine, and mountain environments are complex^4,7,17,18^ and a topic of ongoing investigation. The atmospheric photochemical oxidation of volatile precursor compounds (e.g., fluorotelomer alcohols) is considered a major source of PFAA to remote environments. However, Sha et al.^19^ recently showed that some PFAA can be re-emitted from surface ocean water back into the atmosphere through the generation of sea spray aerosol, which may have significant implications for their hemispheric or global transport. Measurements in snow and air at coastal sites in Antarctica have shown the occurrence of PFAS in this region;^9,20,21^ however, the relative importance of each mechanism should be assessed for individual PFAA and on a site-specific basis.^22^

Once deposited to the snowpack, PFAA are expected to remain there because of their high environmental persistence and low volatility (as conjugate base anions). Subsequent accumulation of snow layers can therefore serve as a natural archive of PFAA concentrations in air, providing a proxy for historic global emissions. A number of studies have followed a similar approach in the Arctic^6,7^ and Tibetan Plateau.^18^ However, ice cores from the high elevation of the East Antarctic plateau are even further removed from source regions, in essence providing a global picture of the atmospheric composition and allowing for the quantitation of PFAA variability and exploration of the mechanisms of global atmospheric transport processes.^23^ The primary aim of this study is therefore, to obtain time trends of PFAA using a dated firn core collected from continental Antarctica to evaluate global usage since the 1960s. In turn, changes in global production as well as the effectiveness of industry and regulatory measures curtailing the production and use of certain chemicals can be assessed.

## 2 Methods

### 2.1 Sample Site and Collection

**Figure 1:**
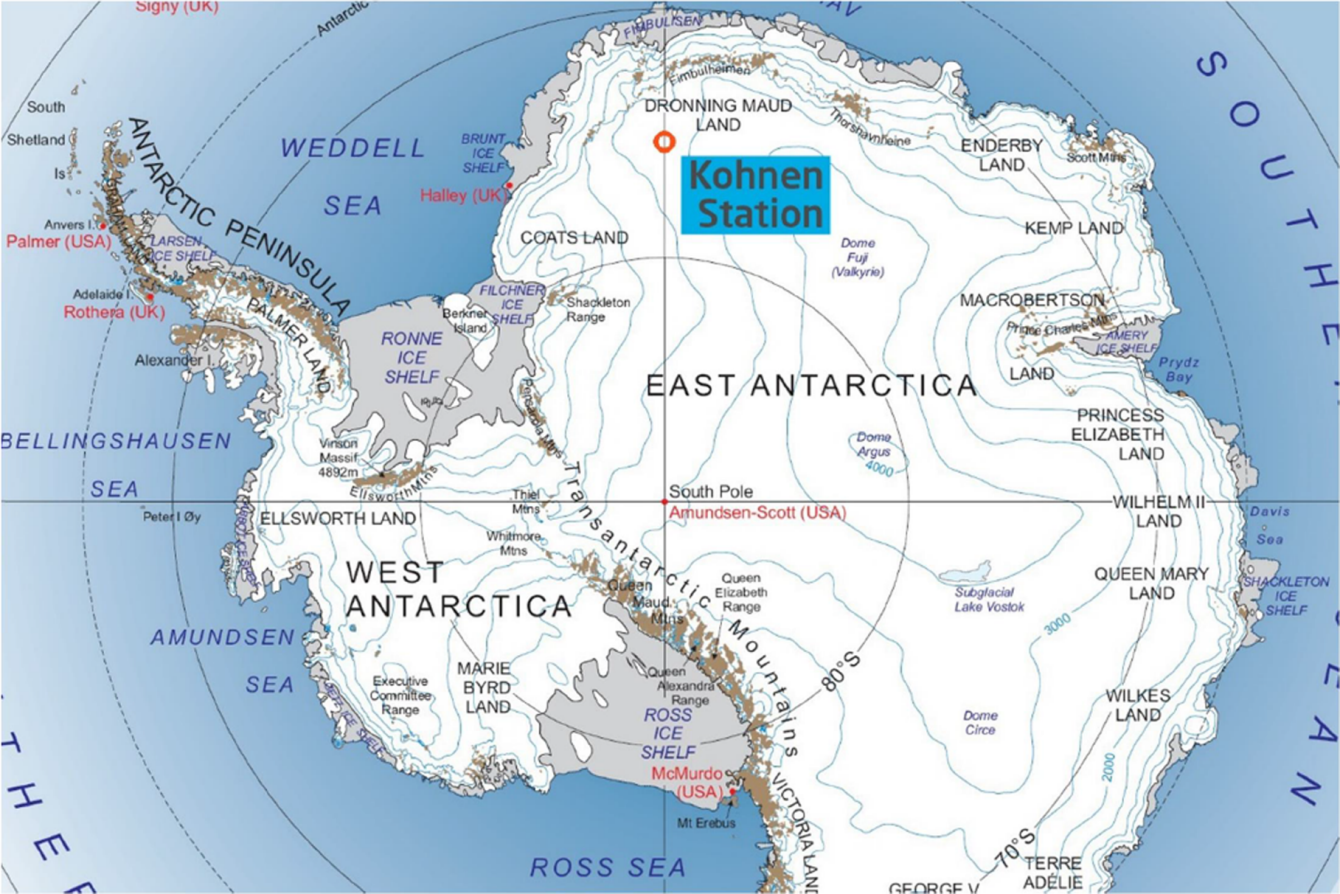
Antarctic land mass with the location of Kohnen station where the firn cores were extracted.^25^

Firn cores were recovered during the “Isotopic Constraints on Past Ozone Layer in Polar Ice” (ISOL-ICE^24^) field season in January 2017 in the clean air sector of Kohnen Research Station, Dronning Maud land, Antarctica (74°59.738′S, 0°05.710′E, 2892 m above the sea level; see Figure 1). Cores analyzed in this study (firn core C = 9.71 m; firn core D = 0.94 m) were collected using a stainless-steel hand drill (96 mm internal diameter). To mitigate potential contamination, handling of the core avoided contact with materials containing fluoropolymers (e.g., Gore-tex). Core sections were wrapped in aluminium foil, placed in polyethylene bags, shipped to the United Kingdom, and stored at −35 °C until further handling.

### 2.2 Dating and Ancillary Measurements

The firn cores were dated using a methodology previously applied to ice core studies in Dronning Maud Land that use sodium (Na) and δD values (Figure S1) to identify annual accumulation layers.^26^ Na concentrations in core “A” were measured continuously (1 mm resolution) by inductively coupled plasma mass spectrometry (ICP-MS).^24^ The seasonality of Na at the EDML site has been constrained using multi-year aerosol observations from the EDML ice core site in which Na concentrations display a broad peak in winter/spring (June–October) and a narrower minimum trough in summer (January).^27^ δD (Los Gatos Research) was measured on 1.5 cm resolution discrete samples of firn cores “C” and “D”. Water isotope values in firn cores “C” and “D” showed excellent agreement, allowing for the comparison of depth scales for the various cores. Figure S2 shows the age-depth model for this study. From 0 to 7 m, annual markers (January) positioned were the summer sodium trough aligned with the summer δD peak. Below 7 m depth, the frequency of the δD cycles decreases with respect to sodium cycles, that is, there are multiple sodium peaks within a depth interval of a single a δD peak. From 7 to 10 m, we therefore used the sodium troughs alone as annual markers. An age uncertainty of ±2 years is estimated at the base of firn core C.

### 2.3 Sample Handling and Extraction

Sample processing was undertaken in a class 1000 clean room facility (British Antarctic Survey, Cambridge) and analyzed in a dedicated PFAS-free laboratory (Helmholtz Zentrum Hereon, Germany). In order to obtain sufficient meltwater for PFAS analysis (>0.5 L), firn core C was cut into discrete layers (*n* = 14), which represented multiple years (*x̅* = 4 years; see Table S1). All samples were placed into sterile polyethylene bags, melted at room temperature, and then transferred to precleaned 2 L polyethylene containers. Each sample was spiked with 400 pg of a mass-labeled internal standard (IS) solution (50 μL of a 8 pg μL^–1^ methanol solution) containing nine isotopically labeled surrogate standards (^13^C_4_-PFBA, ^13^C_2_-PFHxA, ^13^C_4_-PFOA, ^13^C_5_-PFNA, ^13^C_2_-PFDA, ^13^C_2_-PFUnDA, ^13^C_2_-PFDoDA, ^18^O_2_-PFHxS, and ^13^C_4_-PFOS) to assess analytical recovery (%) and correct for substance losses during analysis and matrix effects during the measurement. Detailed information on the target analytes as well as on the standards’ purity and concentration is presented in Table S2.

PFAS were extracted from the samples using a 12-port vacuum manifold system equipped with weak anion exchange cartridges (OASIS WAX, 6 cc, 150 mg sorbent, 30 μm particle size, Waters, USA) that had been preconditioned with 3 mL of formic acid (0.1% v/v), followed by 3 mL of methanol and then 3 mL of water. Samples were loaded at a flow rate of 2–3 drops s^–1^, and a washing step was implemented using 6 mL of SPE-cleaned ultrapure water (18.2 MΩ cm; MilliQ water). Cartridges were mounted with an additional cartridge before being air-dried under vacuum for 40 min to avoid air-borne contamination and remove residual water. Cartridges were stored at −20 °C until further analysis. The target analytes were eluted using 5 mL of methanol, followed by 5 mL of ammonium hydroxide in methanol (0.1% v/v). Finally, the eluates were reduced to 150 μL under nitrogen, and 400 pg of the injection standard ^13^C_8_-PFOA (10 μL of a 40 pg μL^–1^ in methanol) was added with 40 μL of H_2_O to achieve a 80:20 (v/v) methanol:water solution.

### 2.4 Instrumental Analysis

PFAS analysis was undertaken by High-Performance Liquid Chromatography tandem Mass Spectrometry (HPLC–MS/MS) using an HP 1100 LC system (Agilent Technologies, USA) coupled to an API 4000 triple quadrupole mass spectrometer (AB Sciex, USA), operating in negative electrospray ionization mode. The injection volume was 10 μL for all test solutions with chromatographic separation undertaken on a polar-embedded reversed-phase C_18_ separation column (Synergi Fusion-RP C18, 150 mm × 2 mm, particle size 4 μm, pore size 80 Å, Phenomenex, USA). A mobile phase flow of 0.2 mL min^–1^ was set using a gradient elution comprising 2 mM ammonium acetate aqueous solution (A) and 0.05% acetic acid in methanol (B). Detailed information on instrument and compound-specific parameter settings can be found in Tables S3–S6. In brief, a 14-point mixed calibration standard (including all PFAS analytes) was prepared for quantification (80:20 (v/v) methanol:water) in the concentration range from 0 to 25 pg μL^–1^. A linear regression model was utilized and resulted in all analyte calibration curves that were acceptable when *R*^2^ > 0.99. Curves were weighted by applying a factor of 1/*x* to improve accuracy for very low analyte concentrations. All target analytes in the samples were recovery-corrected using a mass-labeled or structurally similar labeled chemical surrogate (e.g., CF_3_(CF_2_)_*n*±1_COOH). Laboratory blanks (*n* = 4) were used to calculate method detection limits (MDL), where MDL = *x̅*_laboratory blank_ + 3σ_laboratory blank_. In cases where analyte concentrations were less than the instrumental limit of detection (LOD) in laboratory blanks, the average concentration in the field blanks (*n* = 2; *x̅*_field blank_; Table S7) was used for individual analytes, where MDL = *x̅*_field blank_ (Table S8). In addition, a subset of samples were screened using HPLC-QToF-MS to confirm the presence of PFAS, particularly for several of the short-chain PFAS, including PFBA (C_4_), for which only one mass transition was monitored by LC–MS/MS (Table S9).

### 2.5 Quality Assurance and Quality Control

Because of the wide use of PFAS and their presence in indoor environments, contamination during sample handling, transport, and storage is possible. To assess any contamination artifact, ice-core sections collected from a previous BAS campaign on the Western Antarctic Peninsula and dated to c.1920 (i.e., prior to the onset of PFAS production in the 1950s), provided useful field blanks (*n* = 3; 1.5 L; with IS) and were analyzed alongside the firn core samples (Table S7 and Figure S3). In addition, laboratory blanks (*n* = 4; with IS; and *n* = 3; without IS) consisting of pre-extracted ultrapure water (*n* = 4; 1.5 L; with IS) were also prepared to assess background PFAS levels associated with laboratory equipment/consumables and processed in an identical manner as the firn core samples (Table S8). Measurement repeatability of PFAS was assessed through duplicate analysis (*n* = 2) of some firn core samples (where sufficient amount of meltwater was available) at two time points within the time series. Reproducibility of PFAS concentrations was also assessed through analysis (*n* = 2) of a separate firn core (Firn core D = 0.94 m) collected to gather further information on PFAS concentration variability in the field.

### 2.6 Data Analysis

Sample concentrations in this study were not blank-corrected because of very low levels of contamination, but samples where analytes fell below method detection limits are highlighted in all figures and tables. Measurement uncertainty for concentrations of each PFAS was estimated using both repeatability (*n* = 2) and reproducibility (*n* = 2) replicates (see eq S1). Dated-firn density measurements (kg m^–3^; Table S10 and Figure S4) was used to calculate average snow accumulation rates (kg m^–2^ yr.^–1^; Table S11 and Figure S5) and annual deposition fluxes of chemicals (ng m^–2^ yr.^–1^; eq S2). Statistical analyses were performed in RStudio (Version 1.1.453; RStudio Team, 2015) using a significance level of α = 0.05. Normality was tested using the Shapiro-Wilks test before further statistical testing followed. Spearman’s rank correlation analysis was subsequently used to assess relationships between PFAS homologs.

## 3 Results and Discussion

### 3.1 Kohnen Station and PFAA

Kohnen Station is a scientific base used to support ice drilling projects and is situated on the East Antarctic Plateau (Dronning Maud Land), located approximately 500 km from the coast at an altitude of 2892 meters above the sea level (see Figure 1 for map). PFAA in snow at Kohnen Station will be influenced by several factors including the prevailing meteorology (i.e. wind direction) and relative proximity to possible chemical sources such as scientific bases^28^ and marine influences.^29^ PFAA were detected in the ice field blanks dated to c. 1920 (see Table S7) with specific chemicals including PFOA (150–2622 pg L^–1^) and PFOS (14–182 pg L^–1^). The presence of these chemicals, particularly in outer core layers of the field blanks, and absence in the firn core drilled at Kohnen Station, reflect contamination of the cores acquired during handling and storage. Nevertheless, the levels of all PFAA in snow at central Dronning Maud Land were well above method detection limits even in snow samples dated well before the station opened in 2001.^30^ Furthermore, we did not detect PFHxS, which has been used as a tracer of direct sources because its volatile precursor compounds were not manufactured significantly over time.^31^ Together, these results demonstrate that PFAA measured in our firn core most likely originate from long-range transport processes and not contamination from handling/storage or from the nearby research station.^9,28^

Time-series data from firn cores can be affected by a number of postdepositional processes,^32,33^ which can alter the chronology of chemical contaminants in the snowpack and therefore result in temporal trends that can reflect local weather events rather than synoptic or even global influences (e.g., melting and snow accumulation rates). However, Kohnen Station is reported to encounter very low annual average air temperature of −46 °C (typically ranging between −15 and −70 °C) with snow accumulation rates 71 ± 21 kg m^–2^ yr.^–1.^^34^ Average snow accumulation rates in this study (snow water equivalent) during the time period under investigation was 69 ± 17 kg m^–2^ yr.^–1^ (Table S11), this result is in excellent agreement with those determined by others, making the core ideal to examine PFAA temporal trends.

The occurrence of PFAA in the remote polar coastal environment has been associated with direct transport through ocean currents^35^ and air, together with volatile precursor compounds with subsequent photochemical oxidation and deposition. It has been proposed that in Antarctica, pollutant advection via ocean currents is minor due to the influence of the Antarctic circumpolar current (ACC), which limits the exchange of relatively polluted waters with the pristine waters of the Southern Ocean.^36^ Nevertheless, Casal et al. (2017) demonstrate that oceanic transport of PFAS from industrial regions to Antarctica can occur and, as a result, this may influence the transport of PFAA directly to remote continental sites, through their association with sea spray aerosol.^19,29^

### 3.2 PFAA Concentrations in Snow

PFAA were detected in all the firn core samples, and the complete data set can be found in Tables S12 and S13. Concentrations of ΣPFAA (C_4_–C_14_) ranged between 137 and 4711 pg L^–1^ (melt water equivalent). Perfluorocarboxylates (PFCA) were the only PFAS that were detected at quantifiable levels with PFBA (C_4_) concentrations over two orders of magnitude higher compared to other PFCA (>95% ΣPFAA). Interestingly, the perfluoroalkane sulfonic acids (PFSA), such as the shorter chain PFBS (C_4_), were not detected in any of the snow samples, and PFOS (C_8_), was below the method detection limits. The concentrations in the present study were compared with other polar and remote locations. In general, the concentrations of PFAA (C_6_–C_14_) at central Dronning Maud Land were akin, albeit lower, to other studies during similar time periods. For example, the concentration of PFOA (C_8_) was ∼120 pg L^–1^ (between 1997 and 1999), compared to approximately 150 and 181 pg L^–1^ in snow from the Tibetan Plateau^18^ and from the Canadian Arctic (Devon ice Cap),^7^ respectively. Similarly, the concentrations of PFOA (C_8_) and other long-chain PFAA in fresh surface snow from coastal Antarctica^21^ in 2015 were much higher (PFOA = 210 pg L^–1^) compared to those reported in this study at central Dronning Maud Land (PFOA = 100 pg L^–1^). Concentrations of PFBA (C_4_) (∼4.4 ng L^–1^ around 2014) in snow were also similar to other studies in remote regions, albeit slightly higher in this study. For example, a previous study^6^ reported a concentration of 1.9 ng PFBA L^–1^ on the Mt. Oxford ice field in Canada in 2014. In some instances there is uncertainty in the reported values of several short-chain PFAA (e.g., PFBA), which is related to isotopically labeled analogues being unavailable at the time of analysis and, in some cases, the use of only one mass transition for compound qualification (see Table S9). An isotopically labeled PFBA was utilized in this study, and its native compound was confirmed through the use of high-resolution mass spectrometry (HRMS). The observation that PFAA levels in the high-altitude remote snowpack of continental Antarctica are generally lower than Arctic or mountainous study sites highlights the remoteness of central Dronning Maud Land. Higher concentrations of some chemicals, such as PFBA, probably reflect differences in atmospheric lifetimes of precursor chemicals and possibly more efficient transport pathways to the continental site (see Section 3.4 Sources and Transport of PFAA).

### 3.3 Time-Series and Depositional Fluxes of PFAA

**Figure 2:**
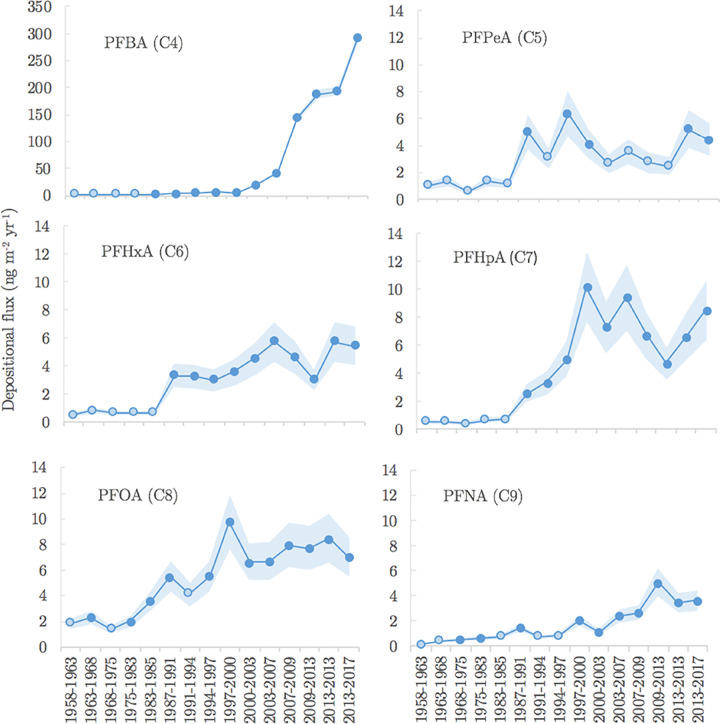
Depositional flux time series of PFCA (C_4_–C_9_) between 1958 and 2017. Open symbols represent those samples that were below method detection limits. Shading indicates estimated concentration uncertainty (±1 s.d.) using repeatability and reproducibility samples.

Figure 2 displays the depositional fluxes time series (ng m^–2^ yr.^–1^) of PFAA (C_4_–C_9_) with the corresponding concentrations summarized in Table S12. For PFBA (C_4_), the depositional flux increased continuously during the studied time period (1958–2017) and showed a rapid increase following the years 2000–2003. The observed trends in the firn core closely matches the time trends observed for PFBA and other short-chain PFCA (≤C_3_) observed by Pickard et al. (2020) in dated firn cores sampled from the Canadian High Arctic.

In contrast to PFBA, depositional flux time-series for other PFAA in the firn core were more variable, although trends corresponded well with those observed in other abiotic and biotic media.^13,37^ Importantly, all PFAA fluxes increased over the same time period. Notably, PFOA (C_8_) showed a sharp increase between 1997 and 2000 and then followed by a decline in 2000–2003. The sharp increase in PFOA occurred prior to the year 2000 when 3 M (a major producer of PFAS) announced historical changes in its PFAS manufacturing and pledged to phase-out production of some chemicals (e.g., PFOS). As a result, many industrial sources located in the US, Europe, and Japan are reported to have also stopped production.^38^ The observed decline of PFOA over the 2000–2003 period is therefore likely to be linked to this announcement through a reduction in use of precursor chemicals that degrade in the environment to form PFOA.

However, our results show that rather than continuing to decline post-2003, the levels of PFOA and other PFCA in Antarctica began to increase again up to 2013. Following the closure of major industrial sites of PFAS production (long-chain PFCA, fluoropolymers, and other PFAS products) across the US, Western Europe, and Japan, geographical production shifted to emerging Asian economies such as China and India.^39^ As such, our results suggest that increasing fluorochemical production in these regions likely offset emission reduction in North America and Europe and account for the higher concentrations observed in the later years of the firn core. Our results are consistent with modeled estimates of global PFCA emissions,^38^ which show that firn cores collected from central Dronning Maud Land can be used to evaluate global elements of PFAS usage patterns. It also highlights the efficient transport of chemicals from industrial regions to remote Antarctic snow, which can occur on timescales on the order of months.

Perfluoroalkyl acids (PFAA) are highly persistent and nonvolatile, particularly as they are present predominantly as their conjugate bases, making chemical losses from the snowpack through volatilization^40^ or degradation of minor significance. Nonetheless, PFAA can be affected by other postdepositional processes such as snowpack aging, precipitation, and/or episodes of meltwater percolation^33^ from the surface during periods of higher temperatures. However, low annual temperatures even during Summer (annual mea *n* = −46 °C)^41^ and well-preserved isotopic profiles with a few visible “ice lenses” (indicative of freeze–thaw activity) provide confidence that the occurrence of such processes during the studied time period were negligible. Furthermore, short-chain PFAA, such as PFBA (C_4_), which display relatively high aqueous solubilities do not show any evidence of postdepositional migration in the snow column with the PFBA temporal profile illustrated in Figure 2.

### 3.4 Sources and Transport of PFAA

While PFAA at remote polar sites like central Dronning Maud Land are expected to have derived primarily from the atmospheric oxidation of gas-phase volatiles followed by wet deposition, concentrations of PFAA in the snow (pg L^–1^) samples were poorly correlated with annual snow accumulation (kg m^–2^ yr.^–1^), suggesting that wet deposition may be of lesser importance compared to dry deposition. PFAA could have also arisen from the direct transport of gas phase and/or particulate-bound PFAA from processes such as aerosol created from the sea surface micro layer.^19^ However, transport of marine aerosols in prevailing air masses affecting central Dronning Maud Land is considered to be relatively small,^27^ and parallel studies have demonstrated very low levels of aerosol-derived particulate matter in the snow.^24^ Moreover, Johansson et al. (2019) showed that aerosol fractions were significantly enriched in only the long-chain PFCA (predominantly >C_9_), which comprised only a small component of the PFAA burden in the snow samples collected in this study. Xie et al. (2020) also suggested that scientific research stations are a source of PFAS contamination; however, the firn coring site in this study was located in a clean air sector away from the Kohnen Station buildings, and our samples revealed no evidence of contamination (see Section 3.2 PFAA concentrations in snow) such as erratic or high levels of PFAS (see Table S12).

The atmospheric photochemical transformation process of PFCA precursor compounds such as fluorotelomer alcohols may occur in the gas phase and/or through heterogeneous reactions occurring on ice-crystal surfaces with subsequent scavenging of the PFAA by snowfall. However, as discussed earlier, poor correlations between PFAA concentrations and snow accumulation (kg m^–2^ yr.^–1^) suggest that snow plays a minor role in the chemical transformation of PFAA precursors. Nevertheless, snow is an effective scavenger of airborne semivolatile organic compounds^40^ including PFAA because of its large surface area and low temperatures, which promote surface sorption. As a result, snow has been shown to play an important role in the transfer of PFCA and PFSA from the atmosphere to remote marine,^42^ alpine,^4^ ice cap,^6,7,15^ and coastal regions of Antarctica.^20,21^ An array of volatile fluorinated precursors^43^ is known to give rise to short- and long-chain PFCA during atmospheric transport^44−46^ and many display sufficient atmospheric lifetimes (i.e., >14 days) to undergo long-range atmospheric transport from the Northern Hemisphere to continental Antarctica. A major class of volatile precursor compounds include fluorotelomer alcohols (FTOH)^47^ with atmospheric photochemical oxidation of n:2FTOH by hydroxyl radicals, resulting in roughly equal proportions of even and odd carbon chain length PFCA homologs consisting of “*n*” and “*n* + 1” carbons.^46^ For example, 6:2FTOH is expected to produce similar molar yields of PFHxA (C_6_) and PFHpA (C_7_). Strong correlations for PFHxA:PFHpA (*r*_s_ = 0.78, *p* < 0.001; *n* = 10) and PFOA:PFNA (*r*_s_ = 0.85, *p* < 0.01; *n* = 11) in firn suggest that these pairs share a common source and may have originated from the photooxidation of 6:2FTOH and 8:2FTOH, respectively (Figure S6). In addition, a depositional even-odd flux ratio of 0.9 ± 0.3 (mean ± 1 s.d.) for PFHxA:PFHpA averaged for the entire time-series (Table S14 and Figure S7) provides further evidence that the presence of PFHxA (C_6_) and PFHpA (C_7_) in the accumulated snow pack is primarily from the photochemical oxidation of 6:2FTOH.

The marked increase in the PFBA flux around the year 2000 may be attributed to the shift in manufacturing from longer chain length PFAS to short-chain chemicals such as the volatile fluorotelomer alcohol, 4:2FTOH, which has been identified in ambient air across Asia.^48^ While some variation in the photochemical product yield from FTOH in the environment is expected,^49^ depositional flux ratios significantly >1 for PFBA:PFPeA (18 ± 27) suggest that 4:2FTOH is actually a minor source of PFBA to central Dronning Maud Land. Hence, other precursors are likely to account for the occurrence of PFBA. Increased production of short-chain perfluorobutane sulfonyl fluoride (PFBSF)-based substances has been reported in the early 2000s, with measurements of *n*-MeFBSA and *n*-MeFBSE^50^ in the coastal atmosphere off the Antarctic continent.^5,8^ However, given the remarkably high concentrations of PFBA in the snow with high depositional flux-ratios and the absence of PFBS (C_4_) in snow, a known degradant of PFBSF-based compounds,^50^ it is unlikely that either 4:2FTOH or PFBSF-based chemicals can account for the marked increases in the concentrations and fluxes of PFBA in recent years. It is plausible that chemicals associated with the direct manufacture of PFBA for unknown uses may also be a source, although one major US producer ceased production in 1998 before concentrations at central Dronning Maud Land began to rise.^51^ Using the average concentration (pg L^–1^) observed in the uppermost part of the firn core (specifically between the years 2013–2017) and scaling up to account for the snow accumulation on the entire Antarctic continent (2100 Gt) modeled by a previous research group^52^ result in an estimated annual deposition of 8892 ± 353 kg of PFBA (see Table S15). This indicates that a significant contemporary global source of PFBA exists, most likely through precursor chemicals that are subsequently transformed to PFBA in the environment. Several CFC (chlorofluorocarbon)-replacement chemicals have been identified as potential precursors to PFBA. One particular group is the hydrofluoroethers (HFEs), which are used as specialized electronic cleaning solvents and heat transfer fluids in refrigerants and in cosmetic applications,^53^ which include HFE-7100 (C_4_F_9_OCH_3_) and HFE-7200 (C_4_F_9_OC_2_H_5_). Both of these are reported to be widely used chemicals^54^ and can lead to the formation of PFBA^51^ and other PFAA^55^ through photochemical oxidation processes in the environment. In 2000, HFEs were submitted to the US EPA for production,^56^ coinciding with the onset of the upward trend of PFBA observed in the firn core in 2000. The atmospheric lifetimes of precursors to PFBA are much longer (several years) for CFC replacements^55^ compared to fluorotelomer precursors (several days), which will result in significantly different abundances of this particular chemical in different regions of the world. A higher [PFBA]:[PFOA] ratio is therefore expected in the Southern Hemisphere (i.e., Antarctica) than in the Northern Hemisphere (i.e., Arctic) where most industrial emissions occur. Our simple assessment for [PFBA]:[PFOA] in Antarctic and Arctic snow^6,7^ was ∼10 and ∼1, respectively. This finding further supports the assertion that CFC replacement chemicals are responsible for high levels of PFBA and other short-chain PFAA at central Dronning Maud Land, Antarctica.

The depositional flux-ratio was also >1 (4.4 ± 2.2) for PFOA:PFNA, which indicates that other chemical precursors in addition to 8:2FTOH may contribute to the presence of PFOA. Various PFASF-based (perfluoroalkane sulfonyl fluoride) compounds such as *n*-alkane perfluorooctane sulfonamides (*n*-FOSA) and n-alkane perfluorooctane sulfonamide ethanols (*n*-FOSE) are known precursors to PFOA, and they have previously been measured in the remote atmosphere in coastal Antarctica.^5,8^ However, the atmospheric oxidation of these compounds are also major sources of PFOS (C_8_), which was on or below detection limits in this study. This could suggest that PFASF-based substances preferentially yield PFCA as degradation products under certain environmental conditions.^45^ Alternatively, PFOA (and other PFCAs) may be formed from chemical intermediates (e.g., perfluoroalkyl aldehydes; PFAL), which are degradation products of fluorotelomer compounds and are also susceptible to long-range transport.^57^ Evidence of other precursors is displayed in Figure S7, which shows a downward trend in the depositional flux ratio of PFOA:PFNA over the time series. While there is some uncertainty and variability in the atmospheric formation of PFCAs from fluorotelomer precursors,^49^ this observation suggests that emissions of precursors that preferentially form PFNA (C_9_) have increased considerably in recent years, relative to PFOA (C_8_). These may have developed as impurities of other FT-based compounds or are produced intentionally (e.g. fluorotelomer olefins; FTO) as intermediates for processing aids in fluoropolymer manufacture, notably polyvinylidene fluoride, which has seen an increase in production.^43^ An increase in indirect emissions of PFNA (C_9_) contradicts emission estimates by Wang et al. (2014b) and therefore highlights the value of using environmental archives, like firn and ice cores, to provide insight into the temporal trends of global emissions.

**Figure 3:**
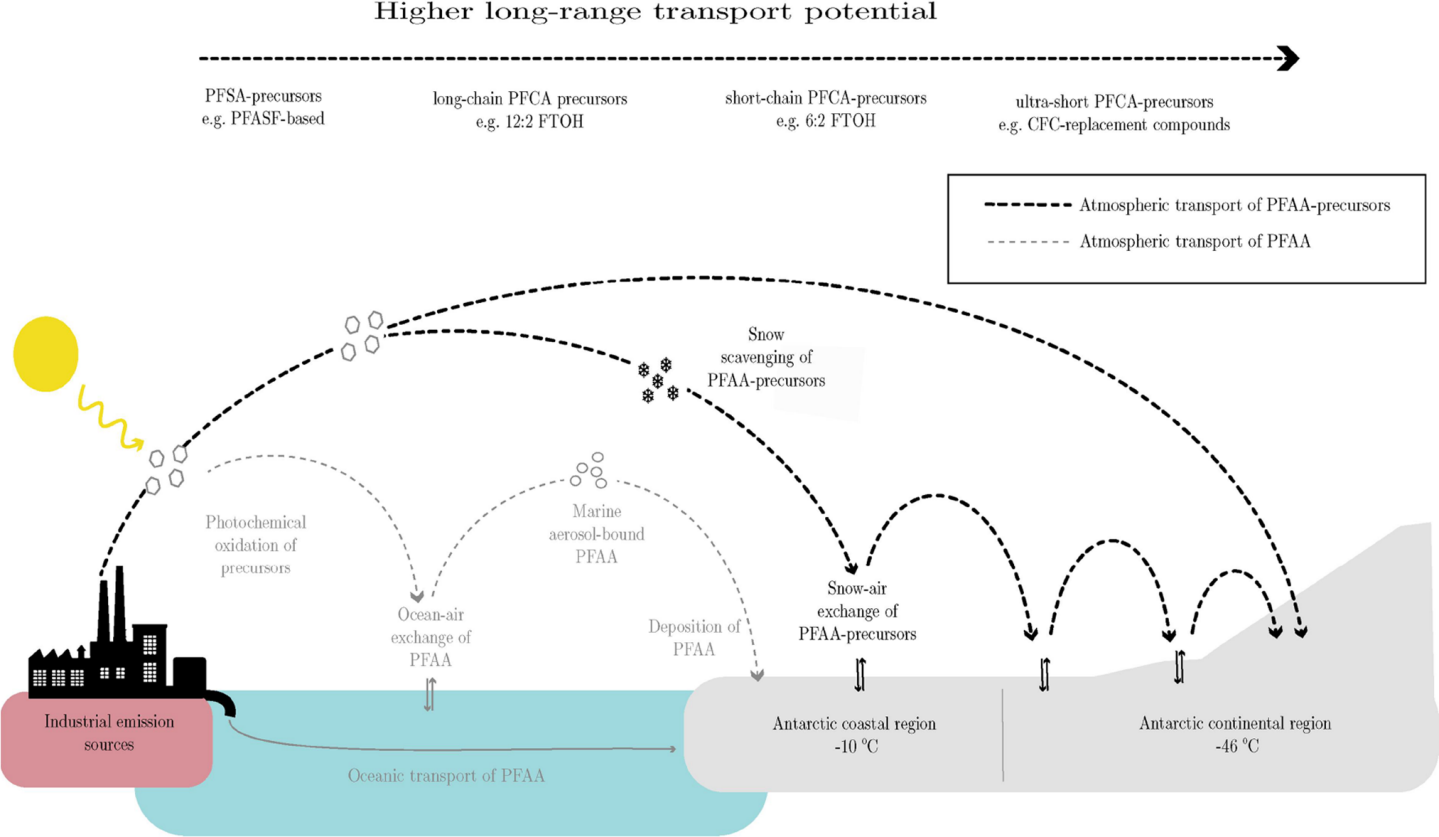
Long-range transport processes of PFAA to Antarctica. Atmospheric emissions of volatile PFAA-precursors undergo hemispheric or global transport and can be photo-oxidized to PFAA, with subsequent deposition and accumulation in the remote snowpack. Higher snowfall in the coastal margins of Antarctica relative to the continental interior will effectively scavenge perfluoroalkane sulfonates and their perfluoroalkane sulfonyl fluoride-based precursors relative to perfluorocarboxylates and their fluorotelomer precursors.

The absence of PFSAs in the firn core is noteworthy, given their measurement in coastal Antarctic snow^21^ and measurement of PFSA-precursors in coastal Antarctic air, which display^5,8^ sufficient atmospheric lifetimes to reach Dronning Maud Land. Some PFAA precursors interact strongly with snow surfaces, altering their long-range atmospheric transport potential.^10^ For example, sorption to snow is significantly greater for PFASF-based compounds compared to FT-based compounds. In turn, this could reduce the travel distance of the PFASF-based compounds and their photodegradates upon encountering cold polar environments (e.g., coastal regions to the higher altitude interior; see Figure 3). Coastal sites at the sea level generally receive greater snowfall, and therefore, PFSAs (and their precursors) are likely to be scavenged more effectively from the atmosphere at Antarctica’s coastal margins, resulting in lower quantities of PFSAs reaching the higher altitude interior.^45^ This explanation may also account for the significantly lower concentrations and lower proportions of long-chain PFCA observed in our study compared to fresh snow deposited in Antarctic coastal margins (e.g., Casal et al., 2017), whereby long-chain precursors (e.g., 12:2FTOH) interact more strongly with snow surfaces than short-chain precursors (e.g., 6:2FTOH).

### 3.5 Global Emissions and Policy Implications

The accumulation of PFAA in snow at central Dronning Maud Land, Antarctica, over the period 1958–2017 appears to be primarily driven from the atmospheric transport and degradation of volatile precursors. The dated firn core in this study is the longest record of PFAS deposition in the remote snowpack and provides a unique PFAA time-series within the Southern Hemisphere. Although there are differences in PFAA concentrations and composition compared with other remote sites around the world, temporal trends and homologue ratios enabled us to determine several broad groups of chemicals that may be contributing PFAA to Antarctica. The high contemporary accumulation fluxes of PFBA is likely due to ongoing use of a high production volume chemical, possibly used to replace CFCs or related compounds rather than for fluoropolymer production.

The Montreal Protocol entered into force in 1987 because of the depletion of stratospheric ozone caused by CFCs. Along with the Kyoto Protocol in 2005, these international policies aim to protect the global environment through responsible chemical management and regulation of various damaging substances. As a result, many chemical companies have been compelled to develop “environmentally friendly” alternatives. As indicated by others,^58^ the Montreal Protocol has, and will, continue to benefit stratospheric ozone levels and climate long into the future. However, the wider environmental impact of such replacements is unknown. Given the high concentrations observed in the remote environment and increasing global demand for heat exchange fluids,^59^ the levels of PFAA are certain to rise in the future.

This study illustrates that firn cores are useful tools that can provide multidecadal records that enable us to better understand the sources and deposition of chemical pollution in the global environment. Our data provide compelling evidence that international legislation and voluntary actions from the chemical industry thus far have been insufficient to reduce the contemporary burden of PFOA and other PFCAs entering the remote Antarctic environment. Given the growing demand for fluorotelomer-based compounds in the foreseeable future,^60^ then deposition of PFAA into the remote environment is likely to continue. In 2019, PFOA was included in the Stockholm Convention, which has also been ratified by China. Thus, it will be useful to examine how global usage patterns develop over the next decade. We propose that more research is conducted with firn and ice core proxies to (i) provide long-term monitoring of PFAS; (ii) identify further chemical PFAA-precursors; and (iii) evaluate the effectiveness of global chemical policies.

## Supporting Information Available

Snow core dating and analytical method information with all quality assurance criteria and full concentration data (PDF)
