# Supplementary material for: Increasing Accumulation of Perfluorocarboxylate Contaminants Revealed in an Antarctic Firn Core (1958–2017)
Source: Environ Sci Technol. 2022 Jul 26;56(16):11246–55. doi: 10.1021/acs.est.2c02592 (PMC9386903; doi:10.1021/acs.est.2c02592)
Supplement: Supplementary file 1 — es2c02592_si_001.pdf [file es2c02592_si_001.pdf]

Supporting Information to:

Increasing accumulation of perfluorocarboxylate contaminants revealed in an Antarctic firn core (1958-2017)

Jack Garnett[1], Crispin Halsall[1*], Holly Winton[2,3], Hanna Joerss[4], Robert Mulvaney[2], Ralf Ebinghaus[4], Markus Frey[2], Anna Jones[2], Amber Leeson[1], Peter Wynn[1]

1 Lancaster Environment Centre, Lancaster University, Lancaster, LA1 4YQ, UK

2 British Antarctic Survey, Cambridge, High Cross, Madingley Road, Cambridge, CB3 0ET, UK

3 Antarctic Research Centre, Victoria University of Wellington, Wellington, 6012, New Zealand

4 Helmholtz-Zentrum Hereon, Max-Planck-Straße 1, 21502 Geesthacht, Germany

Email: c.halsall@lancaster.ac.uk

Contents include:

Pages (S1 – S22)

Figures (S1 – S7)

Tables (S1 – S15)

Equations (S1 – S2)

Analytical methods

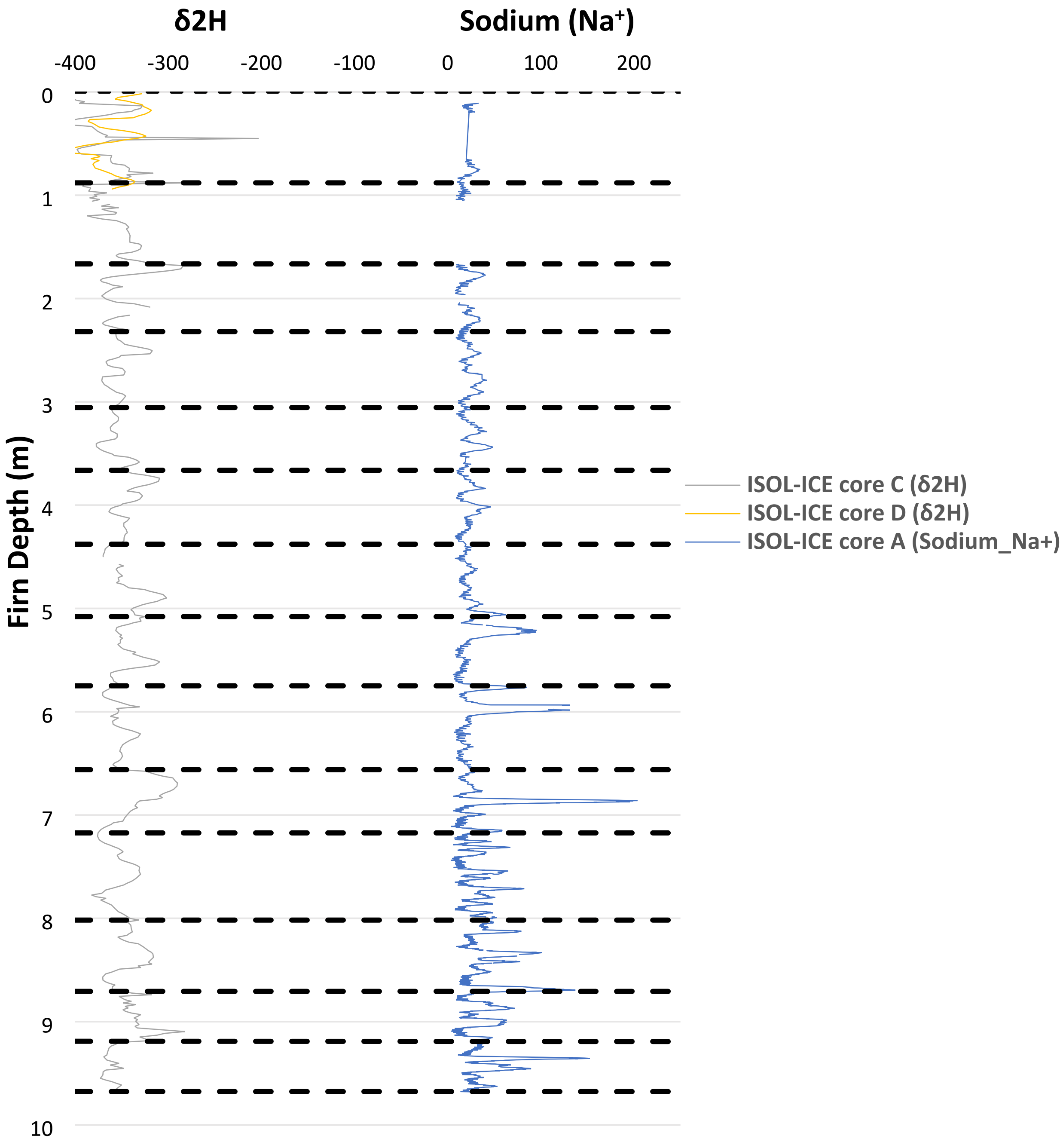

Figure S1: water isotopes and sodium measurements in ice cores for dating.

Firn core dating was achieved following the approach of previous ice core studies in Dronning Maud Land (Göktas et al., 2002). Sodium levels were measured on the main ISOL-ICE core (core A) and δD was measured on 15 mm resolution discrete samples of this firn core (Core

C). Winter peaks of sodium in the main core C aligned with the δD trough in the firn core C confirming the validity of the age scale. Dashed lines represent the depths at which firn core C was sectioned. Some data is missing due to technical issues relating to instrument.

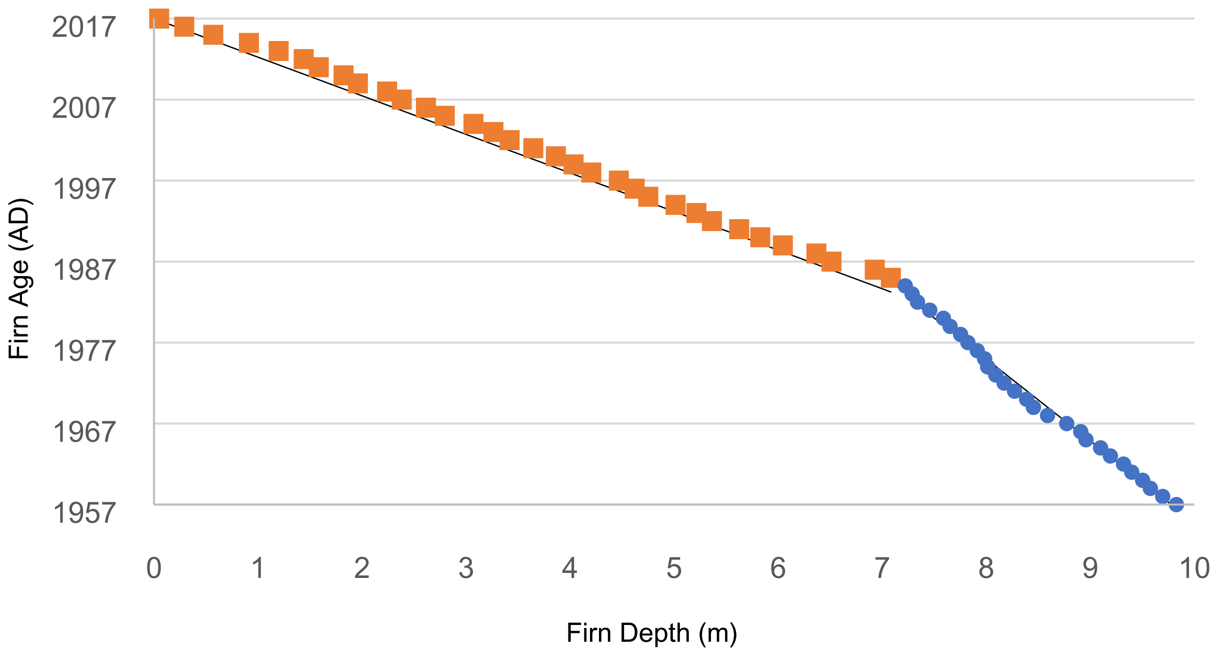

Figure S2: firn age-depth models used in this study

Table S1: Age (AD) and Depth (m) of snow/firn samples measured in this study.

Top depth (m)	Bottom depths (m)	Top year (AD)	Bottom year (AD)	Average year (AD)
0	0.88	2017	2013	2015
*0	0.88	2017	2013	2015
*0.88	0.94	2013	2013	2013
0.88	1.67	2013	2009	2011
1.67	2.32	2009	2007	2008
2.32	3.06	2007	2003	2005
3.06	3.66	2003	2000	2002
3.66	4.38	2000	1997	1999
4.38	5.08	1997	1994	1996
5.08	5.75	1994	1991	1993
5.75	6.56	1991	1987	1989
6.56	7.17	1987	1985	1986
7.17	8.02	1985	1975	1980
8.02	8.71	1975	1968	1971
8.71	9.19	1968	1963	1965
9.19	9.71	1963	1958	1960

Firn samples (ISOL-ICE core C) in this study were dated by applying different age-depth models for firn at 0 – 7 m ($y = -4.5086x + 2017$) and 7 – 10 m ($y = -10.108x + 2055.8$). * Represents samples obtained from ISOL-ICE core D which were used for reproducibility samples (0 – 0.94 m).

Table S2: Overview of analytical standards, CAS numbers, the standard suppliers, purity and concentration/amount

Acronym	Analytical standard	CAS number	Supplier, purity and concentration/amount
PFBA	perfluoro-*n*-butanoic acid	375-22-4 (acid)	PFC-MXA (mixture)<br>Wellington Laboratories,<br>> 98 %<br>2.0 µg/mL ± 5 %<br>of the single compounds
PFPeA	perfluoro-*n*-pentanoic acid	2706-90-3 (acid)	
PFHxA	perfluoro-*n*-hexanoic acid	307-24-4 (acid)	
PFHpA	perfluoro-*n*-heptanoic acid	375-85-9 (acid)	
PFOA	perfluoro-*n*-octanoic acid	335-67-1 (acid)	
PFNA	perfluoro-*n*-nonanoic acid	375-95-1 (acid)	
PFDA	perfluoro-*n*-decanoic acid	335-76-2 (acid)	
PFUnDA	perfluoro-*n*-undecanoic acid	2058-94-8 (acid)	
PFDoDA	perfluoro-*n*-dodecanoic acid	307-55-1 (acid)	
PFTrDA	perfluoro-*n*-tridecanoic acid	72629-94-8 (acid)	
PFTeDA	perfluoro-n-tetradecanoic acid	376-06-7 (acid)	
PFBS	potassium perfluoro-*n*-butanesulfonate	29420-49-3 (K+ salt)<br>375-73-5 (acid)	PFS-MXA (mixture)<br>Wellington Laboratories,<br>> 98 %<br>2.0 µg/mL ± 5 %<br>of the single compounds
PFHxS	sodium perfluoro-*n*-hexanesulfonate	82382-12-5 (Na+ salt)<br>355-46-4 (acid)	
PFHpS	sodium perfluoro-*n*-heptanesulfonate	22767-50-6 (Na+ salt)<br>375-92-8 (acid)	
PFOS	sodium perfluoro-*n*-octanesulfonate	4021-47-0 (Na+ salt)<br>1763-23-1 (acid)	
PFDS	sodium perfluoro-*n*-decanesulfonate	2806-15-7 (Na+ salt)<br>335-77-3 (acid)	
13C4-PFBA	perfluoro-*n*-[13C4]-butanoic acid	-	MPFAC-MXA (mixture)<br>Wellington Laboratories, > 98 %,<br>2.0 µg/mL ± 5 %<br>of the single compounds
13C2-PFHxA	perfluoro-*n*-[1,2-13C2]-hexanoic acid	-	
13C4-PFOA	perfluoro-*n*-[1,2,3,4-13C4]-octanoic acid	-	
13C5-PFNA	perfluoro-*n*-[1,2,3,4,5-13C5]-nonanoic acid	-	
13C2-PFDA	perfluoro-*n*-[1,2-13C2]-decanoic acid	-	
13C2-PFUnDA	perfluoro-*n*-[1,2-13C2]-undecanoic acid	-	
13C2-PFDoDA	perfluoro-*n*-[1,2-13C2]-dodecanoic acid	-	
18O2-PFHxS	sodium perfluorohexane-*n*-[18O2]-sulfonate	-	

13C4-PFOS	sodium perfluoro-*n*-[1,2,3,4-13C4]-octanesulfonate	-	
13C8-PFOA	perfluoro-[13C8]-octanoic acid (injection standard)	-	Wellington Laboratories, > 98 %, (50 ± 2.5) µg/mL

Target PFAS included 11 PFCA (C_4 to C_{14}), five PFSA ($C_{4,}$ $C_{6,}$ $C_{7,}$ $C_{8,}$ C_{10})

Table S3: Overview of the components and the compound-independent parameter settings for the LC-MS/MS analysis of PFAS.

Liquid chromatography					
Component	type (manufacturer, country)				
Binary pump	HP 1100 LC binary pump G1312 (Agilent, USA)				
Autosampler	HP 1100 LC autosampler G1313 (Agilent, USA)				
Analytical column	Synergi Fusion-RP: polar embedded C18 phase with trimethylsilyl endcapping, 150 mm x 2 mm, particle size 4 µm, pore size 80 Å (Phenomenex, USA)				
Guard column	SecurityGuard cartridge for Fusion-RP HPLC columns, 4 mm x 2 mm (Phenomenex, USA)				
Software	Analyst 1.5 (AB Sciex, USA)				
parameter	setting				
injection volume	10 µL (needle rinsed twice with methanol before injection)				
column temperature	30 °C				
flow rate	0.2 mL/min				
mobile phases	A: 2 mM ammonium acetate aqueous solution<br>B: 0.05 % acetic acid in methanol				
sample/standard solvent	methanol:water 80:20 (v/v)				
gradient	time [min]	A [%]	B [%]	note	
	–8	70	30	equilibration	
	0	70	30		
	3	30	70		
	29	10	90		
	31	0	100	purging	
	45	0	100		
tandem mass spectrometry					
component	type (manufacturer, country)				
instrument	API 4000 triple quadrupole mass spectrometer (AB Sciex, USA)				
ion source	Turbo V Ion Source (AB Sciex, USA)				
software	Analyst 1.5 (AB Sciex, USA)				
parameter	settings				
ionization	electrospray ionization (ESI) in negative mode				

ion spray voltage	–4500 V
source temperature	400 °C
gas 1 (nebulizer gas)	N2, 4.2 bar
gas 2 (heater gas)	N2, 2.8 bar
curtain gas	N2, 1.0 bar
collision gas	N2, 0.6 bar
scan type	Scheduled Multiple Reaction Monitoring (MRM)<br>retention time window: 180 s, target scan time: 2 s

Table S4: Compound-specific parameter settings for the LC-MS/MS analysis of native PFAS.

acronym analyte	t_R [min][1]	molecular formula precursor ion	mass transition [m/z][2]	transition-specific parameters [V][3]			
				DP	EP	CE	CXP
PFBA	6.1	[C4F7O2]–	213>169*	–30	–5	–13	–9
PFPeA	9.6	[C5F9O2]–	263>219*	–26	–4	–12	–13
PFHxA	10.4	[C6F11O2]–	313>269*	–28	–4	–13	–16
			313>119	–28	–4	–30	–5
PFHpA	11.1	[C7F13O2]–	363>319*	–29	–4	–14	–19
			363>169	–29	–4	–25	–8
PFOA	11.9	[C8F15O2]–	413>369*	–24	–4	–15	–8
			413>169	–24	–4	–28	–8
PFNA	12.9	[C9F17O2]–	463>419*	–34	–4	–15	–9
			463>219	–34	–4	–24	–12
PFDA	14.2	[C10F19O2]–	513>469*	–35	–6	–15	–11
			513>219	–35	–6	–29	–12
PFUnDA	15.9	[C11F21O2]–	563>519*	–35	–5	–17	–13
			563>169	–35	–5	–37	–8
PFDoDA	18.0	[C12F23O2]–	613>569*	–38	–9	–17	–15
			613>169	–38	–9	–38	–8
PFTrDA	20.3	[C13F25O2]–	663>619*	–39	–8	–18	–14
			663>169	–39	–8	–41	–8
PFTeDA	22.7	[C14F27O2]–	713>669*	–36	–9	–22	–15
			713>169	–36	–9	–40	–8
PFBS	9.7	[C4F9O3S]–	299>99*	–66	–12	–42	–16
			299>80	–66	–12	–60	–2
PFHxS	11.1	[C6F13O3S]–	399>99	–70	–14	–50	–15
			399>80*	–70	–14	–66	–2
PFHpS	11.8	[C7F15O3S]–	449>99	–80	–12	–61	–16
			449>80*	–80	–12	–85	–2
PFOS	12.8	[C8F17O3S]–	499>99	–73	–12	–74	–17
			499>80*	–73	–12	–90	–2
PFDS	15.8	[C10F21O3S]–	599>99	–80	–14	–62	–3
			599>80*	–80	–14	–90	–2

[1] The column provides exemplary retention times. They changed in dependence of the age of the column and were determined before each measurement sequence in a non-scheduled MRM run.
[2] Asterisks mark the product ion that was used as quantifier, whereas the second product ion was used as qualifier.
[3] Optimized mass spectrometric parameters include the declustering potential (DP), the entrance potential (EP), the collision energy (CE) and the cell exit potential (CXP).

Table S5: Compound-specific parameter settings for the LC-MS/MS analysis of the internal standards used for PFAS quantification. 13C8-PFOA was added as injection standard.

acronym analyte	tR [min]	molecular formula precursor ion	mass transitions [m/z]	transition-specific parameters [V]			
				DP	EP	CE	CXP
13C4-PFBA	6.1	[13C4F7O2]–	217>172*	–22	–4	–13	–9
13C2-PFHxA	10.4	[13C212C4F11O2]–	315>270*	–23	–6	–12	–16
			315>120	–23	–6	–31	–4
13C4-PFOA	11.9	[13C412C4F15O2]–	417>372*	–32	–4	–13	–8
			417>169	–32	–4	–27	–8
13C8-PFOA	11.9	[13C8F15O2]–	421>376*	–25	–6	–14	–8
			421>172	–25	–6	–26	–8
13C5-PFNA	12.9	[13C512C4F17O2]–	468>423*	–30	–7	–14	–10
			468>223	–30	–7	–24	–12
13C2-PFDA	14.2	[13C212C8F19O2]–	515>470*	–39	–6	–16	–10
			515>220	–39	–6	–26	–12
13C2-PFUnDA	15.9	[13C212C9F21O2]–	565>520*	–33	–6	–16	–13
			565>169	–33	–6	–34	–8
13C2-PFDoDA	18.0	[13C212C10F23O2]–	615>570*	–38	–9	–17	–15
			615>169	–38	–9	–41	–8
18O2-PFHxS	11.1	[C6F1318O216OS]–	403>103	–82	–10	–55	–4
			403>84*	–82	–10	–79	–2
13C4-PFOS	12.8	[13C412C4F17O3S]–	503>99	–65	–12	–64	–4
			503>80*	–65	–12	–92	–2

Table S6: Overview of the components and parameter settings for the LC-QToF-MS analysis to confirm the identity of PFAS, for which only one mass transition was monitored by LC-MS/MS.

liquid chromatography				
component	type			
system	1290 Infinity II UHPLC (Agilent, USA)			
analytical column	Zorbax Eclipse Plus C18, 2.1 mm x 100 mm, particle size: 1.8 µm, pore size: 95 Å (Agilent, USA)			
software	Mass Hunter, version 10.0 (Agilent, USA)			
parameter	setting			
injection volume	10 µL (needle wash: 20 s flush port, methanol)			
column temperature	30 °C			
flow rate	0.2 mL/min			
mobile phases	A: 2 mM ammonium acetate aqueous solution<br>B: methanol			
gradient	time [min]	A [%]	B [%]	
	0	90	10	
	10	2	98	
	15	2	98	
	15.1	90	10	
high resolution mass spectrometry				
component	type (manufacturer, country)			
instrument	6546 QToF-MS (Agilent, USA)			
software	Mass Hunter, version 10.0 (Agilent, USA)			
instrumental settings				
ionization	electrospray ionization (ESI) in negative mode			
gas temperature	200 °C			
gas flow	12 L/min			
nebulizer gas	45 psig			
sheath gas temperature	350 °C			
sheath gas flow	11 L/min			
mass spectrometric analysis settings				
mode	All Ions (data-independent acquisition mode; all ions are fragmented without a specific isolation of a precursor ion in the			

	first mass analyser)		
mass range	m/z 50–1000		
scan rate	6 spectra/s		
scan segments	#	collision energy [eV]	
	1	0	
	2	10	
	3	40	
reference masses	*m/z* 112.98558700; 980.01637500		

Table S7: Concentrations of PFAAs (pg L^{-1}) in field blank

Sample Name	PFBA	PFPeA	PFHxA	PFHpA	PFOA	PFNA	PFDA	PFUnDA	PFDoDA	PFTrDA	PFTeDA	PFBS	PFHxS	PFHpS	PFOS	PFDS
Interior (Field_blank_1)	<LOD	40.4	18.3	24.5	149.9	9.2	22.1	4.9	11.6	<LOD	2.0	<LOD	<LOD	<LOD	13.9	<LOD
Middle (Field_blank_2)	<LOD	32.9	24.1	28.6	197.2	11.8	32.6	5.8	13.7	1.8	2.8	<LOD	<LOD	<LOD	23.2	<LOD
Exterior (Field_blank_3)	<LOD	113.0	160.1	184.7	2622.8	88.3	351.7	47.4	132.6	8.3	19.8	117.9	13.9	<LOD	181.7	<LOD

We screened a firn core for PFAS that had been acquired from the Antarctic Peninsula and dated to around 1920s (predated the onset of PFAS manufacture in 1950s) to determine whether firn cores that were collected during historical sampling campaigns (now in storage) were viable for temporal PFAS studies. PFAS have now been in-use for several decades within various garments (e.g. gloves) as waterproofing agents and therefore contact with these items during their extraction in the field and/or subsequent handling/storage may serve as a source of contamination thereby compromising their integrity. We cut three samples that consisted of inner, middle and outer edges (see schematic below) and subject each melted sample to PFAS analysis. Concentrations of PFAS in the external samples were significantly higher than inner layers, which suggests that contamination with PFAS at some stage after extraction had occurred with possible diffusion to inner layers. Hence, caution is advised when working with firn cores obtained from historical campaigns, ensuring sufficient outer edge of cores is removed to eliminate possible contamination.

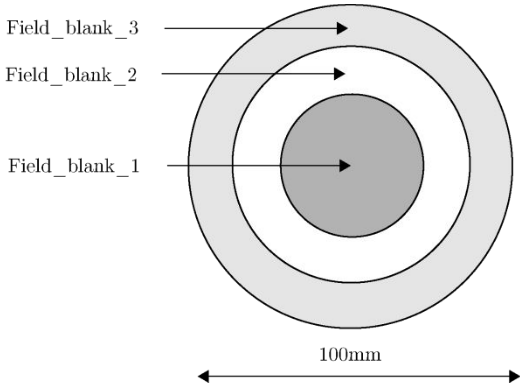

Figure S3: cross section of ice core used as field blanks

Table S8: Quality control/quality assurance in this study.

Native chemical	PFBA	PFPeA	PFHxA	PFHpA	PFOA	PFNA	PFDA	PFUnDA	PFDoDA	PFTrDA	PFTeDA	PFBS	PFHxS	PFHpS	PFOS	PFDS
$\bar{x}_{lab\ blank}$ (n=4) [pg L^{-1}]	27.5	37.4	20.9	14.9	37.0	<LOD	<LOD	<LOD	<LOD	<LOD	<LOD	<LOD	<LOD	<LOD	15.9	<LOD
$SD_{lab\ blank}$ (n=4) [pg L^{-1}]	2.6	3.0	2.5	3.3	4.7	<LOD	n/a	n/a	n/a	n/a	n/a	<LOD	<LOD	<LOD	7.9	<LOD
$\bar{x}_{Field\ blank}$ (n=2) [pg L^{-1}]	<LOD	36.7	21.2	26.6	173.6	10.5	27.4	5.4	12.7	0.9	2.4	<LOD	<LOD	<LOD	18.6	<LOD
$MDL = \bar{x}_{lab\ blank} + 3 \cdot SD_{lab\ blank}$ [pg L^{-1}]	35.4	46.5	28.5	24.8	51.2	*10.5	*27.4	*5.4	*12.7	*0.9	*2.4	<LOD	<LOD	<LOD	39.6	<LOD
% of samples >MDL (n=18)	72	39	67	61	78	72	6	22	6	22	6	0	0	0	11	0
Internal standard (IS)	13C4-PFBA	13C2-PFHxA		13C4-PFOA		13C5-PFNA	13C2-PFDA	13C2-PFUnDA	13C2-PFDoDA			18O2-PFHxS			13C4-PFOS	

Quality control samples were taken to ensure reported chemical concentrations are accurate. Field blanks consisted of a firn core that had its outer surface removed to avoid possible contamination from handling during extraction/storage (See Table S4). Asterisk (*) denotes analytes that have had their MDLs determined using field blank samples as described in methodology. Limit of Detection is defined as the lowest detectable amount of an analyte in the laboratory blanks.

Table S9: Identity confirmation of PFBA using LC-QToF-MS in firn sample dated to 2009 - 2013.

	calibration standard	Firn sample (2009 - 2013)
precursor ion [C4F7O2]–		
theoretical mass	*m/z* 213.98648 (neutral mass)	
observed mass	*m/z* 213.98649	*m/z* 213.98638
mass error [ppm]	0.08	-0.46
fragment ion [C3F7]-		
theoretical mass	*m/z* 168.98937	
observed mass	*m/z* 168.98932	*m/z* 168.98937
mass error [ppm]	-0.29	-0.03
pattern of additional fragments		

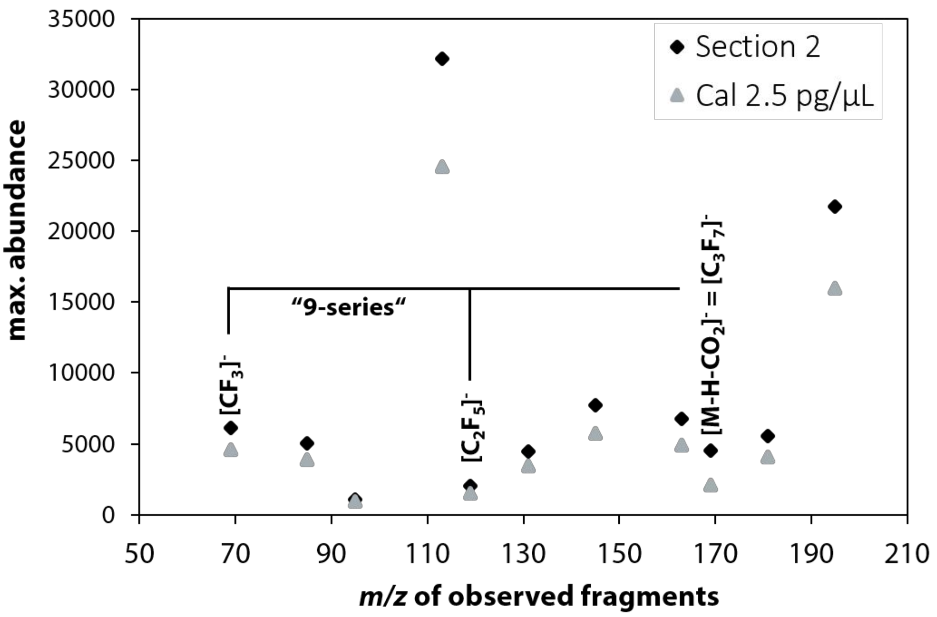

For unequivocal identification, at least two mass transitions by compound have to be monitored by LC-MS/MS. However, for PFBA and PFPeA, only one product ion with high enough intensity was identified when developing the LC-MS/MS method (see Table S4). This is consistent with other scientific publications (Gremmel et al., 2017, Munoz et al., 2015) and norms for the analysis of PFAS (e.g. DIN norm 38407-42:2011-03). To confirm the identity of the substances concerned, standards and selected samples were additionally analysed by high resolution mass spectrometry in this study.

In addition to the retention time and mass transition measured by LC-MS/MS, the exact mass of the precursor ion (mass error << 5 ppm), the exact mass of the selected fragment ion [C3F7]-

(mass error << 5 ppm) and the presence of several additional fragment ions with a comparable abundance pattern in the calibration standard and the sample measured by LC-QToF-MS confirm the identity of PFBA in the sample. It has to be noted that the high resolution of the instrument is compromised with a lower sensitivity compared to the LC-MS/MS measurement (~ factor 10). Consequently, PFBA was not confirmed in samples and standards with low concentrations.

Equation S1: Error propagation used to assess chemical concentration uncertainty

$$\text{Measurement uncertainty } (\%) = \sqrt{\left[\frac{\sigma_{\text{ repeatability } samples}}{\text{x}_{\text{repeatability } samples}}\right]^2 + \left[\frac{\sigma_{\text{ reproducibility } samples}}{\text{x}_{\text{reproducibility } samples}}\right]^2}$$

Where; σ = standard deviation and $\bar{x}$ = mean

Sample variability in PFASs concentrations originates from laboratory analysis and small-scale variations in snow concentrations in the field. Due to the limited number of laboratory (*n*=2) and field (*n*=2) replicates, a conservative approach to assess the total uncertainty associated with each PFAA was adopted using the above formula.

Table S10: Dated firn-density measurements made on snow core (ISOL-ICE core B at Kohnen Station.

Dated firn	Firn Density ($kg\ m^{-3}$)
*2013-2017	311
2013-2013	320
2009-2013	326
2007-2009	339
2003-2007	351
2000-2003	363
1997-2000	374
1994-1997	387
1991-1994	399
1987-1991	412
1983-1985	424
1975-1983	437
1968-1975	451
1963-1968	461
1958-1963	470

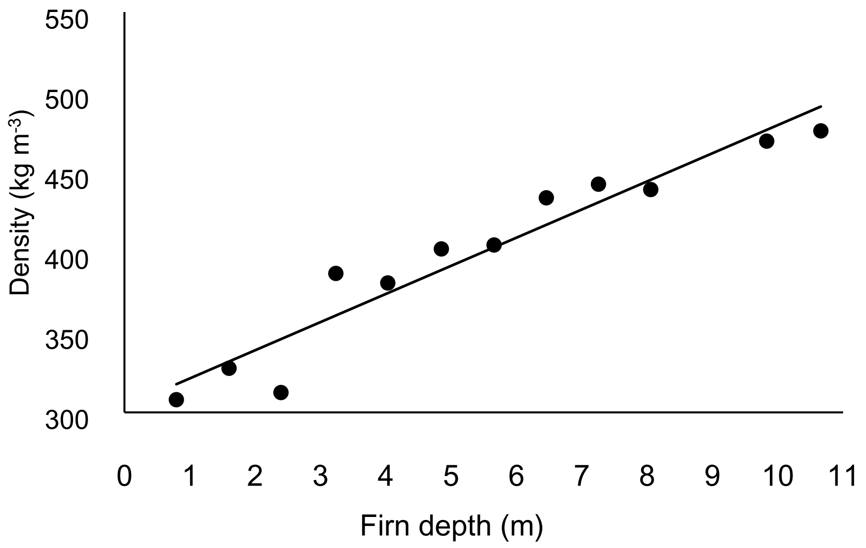

Figure S4: A depth-density model (y = 17.576x + 303.63) from a parallel shallow core (ISOL-ICE core B) was applied to samples in this core

To avoid possible PFAS contamination arising from contact with surfaces, and to preserve the volume of snow meltwater needed for PFAS analysis, firn density was derived from a second shallow snow core that was manually drilled at Kohnen Station during the ISOL-Ice campaign in 2017. Density measurements were determined gravimetrically and a linear regression (See Figure 1) was subsequently applied to derive a density-depth scale for the firn core in this study (Firn core C).

Dated-firn	Snow accumulation rate (kg m^{-2} yr^{-1})
*2013-2017	69
2013-2013	64
2009-2013	72
2007-2009	75
2003-2007	78
2000-2003	80
1997-2000	83
1994-1997	86
1991-1994	88
1987-1991	91
1983-1985	63
1975-1983	43
1968-1975	45

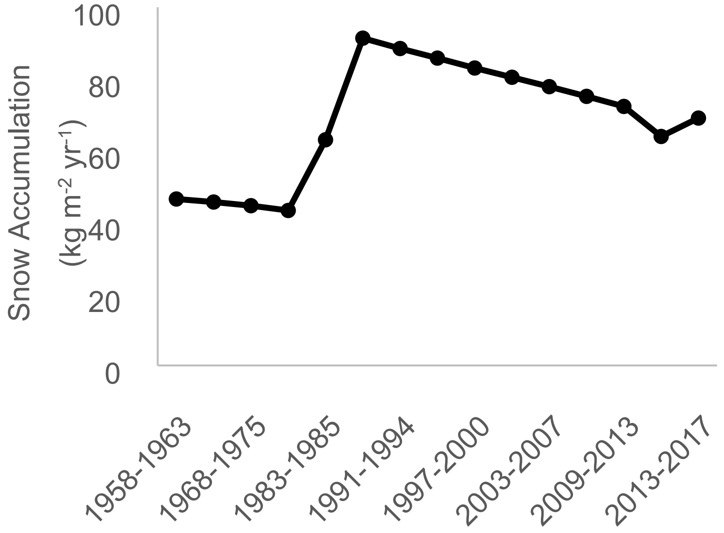

Figure S5: Snow accumulation at central Dronning Maud Land (Kohnen Station), Antarctica

1963-1968	46
1958-1963	46
	Mean (± 1.s.d.) = 69 ± 17

Table S11: Snow accumulation rates at central Dronning Maud Land (Kohnen Station), Antarctica

Equation S2: Average annual of amount of chemical deposited

$$\text{Average annual depositional flux } \left(\text{ng m}^{-2}\text{ yr}^{-1}\right) =$$

$$\left[\frac{\text{Firn density } \left(\text{kg m}^{-3}\right) \text{ x core section length (m) x sample concentration } \left(\text{ng L}^{-1}\right)}{\text{accumulation period (yr)}}\right]$$

Where 1 L of water = 1 kg of water

Firn density can be found in Table S10. Core section lengths and accumulation period can be determined from Table S1.

. Table S12: Concentration of PFAS in dated-firn samples (pg L^{-1})

	PFBA	PFPeA	PFHxA	PFHpA	PFOA	PFNA	PFDA	PFUnDA	PFDoDA	PFTrDA	PFTeDA	PFBS	PFHxS	PFHpS	PFOS	PFDS
1958-1963	25.5	22.6	12.8	12.7	39.5	4.5	7.3	<LOD	<LOD	<LOD	<LOD	<LOD	<LOD	<LOD	12.3	<LOD
1963-1968	26.6	29.1	19.1	13.3	51.7	9.0	7.0	<LOD	<LOD	<LOD	<LOD	<LOD	<LOD	<LOD	14.4	<LOD
1968-1975	26.7	13.5	15.7	10.4	30.0	12.1	32.6	10.7	20.7	4.6	10.7	<LOD	<LOD	<LOD	10.2	<LOD
1975-1983	32.2	27.8	8.6	12.8	37.8	14.3	2.8	8.5	<LOD	3.5	1.2	<LOD	<LOD	<LOD	9.9	<LOD
1975-1983	33.3	35.1	21.6	20.1	57.4	15.2	3.0	4.9	<LOD	2.6	2.4	<LOD	<LOD	<LOD	16.8	<LOD
1983-1985	39.3	17.8	10.3	12.2	56.7	7.6	5.9	2.5	4.6	1.7	1.6	<LOD	<LOD	<LOD	12.4	<LOD
1987-1991	40.2	47.3	31.9	23.6	52.8	17.6	<LOD	7.7	<LOD	<LOD	<LOD	<LOD	<LOD	<LOD	16.1	<LOD
1987-1991	41.9	62.3	40.8	32.9	67.5	14.5	<LOD	<LOD	<LOD	<LOD	<LOD	<LOD	<LOD	<LOD	17.3	<LOD
1991-1994	48.8	34.4	36.8	38.4	46.7	8.0	3.3	4.0	3.2	<LOD	0.9	<LOD	<LOD	<LOD	12.6	<LOD
1994-1997	71.8	74.5	34.9	58.6	64.4	9.9	4.7	3.2	3.6	<LOD	<LOD	<LOD	<LOD	<LOD	13.1	<LOD
1997-2000	57.0	49.2	43.0	122.1	117.8	24.7	<LOD	<LOD	<LOD	<LOD	<LOD	<LOD	<LOD	<LOD	16.7	<LOD
2000-2003	241.2	33.2	56.2	90.9	82.8	14.4	<LOD	3.7	3.4	<LOD	<LOD	<LOD	<LOD	<LOD	17.0	<LOD
2003-2007	541.8	45.2	73.6	120.9	86.1	30.9	<LOD	<LOD	<LOD	<LOD	<LOD	<LOD	<LOD	<LOD	<LOD	<LOD
2007-2009	1920.3	36.3	61.7	89.5	106.2	35.5	7.7	2.6	<LOD	<LOD	<LOD	<LOD	<LOD	<LOD	14.4	<LOD
2009-2013	2607.7	34.3	41.8	65.4	107.0	70.4	9.4	4.0	<LOD	<LOD	1.3	<LOD	<LOD	<LOD	14.1	<LOD
2013-2013	3027.4	81.7	89.7	93.5	132.6	54.3	<LOD	<LOD	<LOD	<LOD	<LOD	<LOD	<LOD	<LOD	52.6	<LOD
2013-2017	4147.0	73.0	81.8	122.9	96.4	46.0	<LOD	<LOD	<LOD	<LOD	<LOD	<LOD	<LOD	<LOD	30.8	<LOD
2013-2017	4321.4	55.6	75.5	123.8	108.0	60.3	19.5	6.8	5.9	<LOD	2.1	<LOD	<LOD	<LOD	11.1	<LOD

Red values indicate those analytes that were below method detection limits

Table S13: Depositional fluxes of PFAS in Antarctica (ng m^{-2} yr^{-1})

	PFBA	PFPeA	PFHxA	PFHpA	PFOA	PFNA	PFDA	PFUnDA	PFDoDA	PFTrDA	PFTeDA	PFBS	PFHxS	PFHpS	PFOS	PFDS
1958-1963	1.3	1.1	0.6	0.6	2.0	0.2	0.4	<LOD	<LOD	<LOD	<LOD	<LOD	<LOD	<LOD	0.6	<LOD
1963-1968	1.6	1.7	1.1	0.8	3.1	0.5	0.4	<LOD	<LOD	<LOD	<LOD	<LOD	<LOD	<LOD	0.9	<LOD
1968-1975	1.4	0.7	0.8	0.6	1.6	0.7	1.8	0.6	1.1	0.2	0.6	<LOD	<LOD	<LOD	0.5	<LOD
1975-1983	1.3	1.1	0.3	0.5	1.5	0.6	0.1	0.3	<LOD	0.1	0.0	<LOD	<LOD	<LOD	0.4	<LOD
1975-1983	1.3	1.4	0.9	0.8	2.3	0.6	0.1	0.2	<LOD	0.1	0.1	<LOD	<LOD	<LOD	0.7	<LOD
1983-1985	2.6	1.2	0.7	0.8	3.8	0.5	0.4	0.2	0.3	0.1	0.1	<LOD	<LOD	<LOD	0.8	<LOD
1987-1991	3.3	3.8	2.6	1.9	4.3	1.4	<LOD	0.6	<LOD	<LOD	<LOD	<LOD	<LOD	<LOD	1.3	<LOD
1987-1991	3.4	5.0	3.3	2.7	5.5	1.2	<LOD	<LOD	<LOD	<LOD	<LOD	<LOD	<LOD	<LOD	1.4	<LOD
1991-1994	4.5	3.1	3.4	3.5	4.3	0.7	0.3	0.4	0.3	<LOD	0.1	<LOD	<LOD	<LOD	1.2	<LOD
1994-1997	4.6	4.7	2.2	3.7	4.1	0.6	0.3	0.2	0.2	<LOD	<LOD	<LOD	<LOD	<LOD	0.8	<LOD
1997-2000	6.1	5.3	4.6	13.1	12.6	2.7	<LOD	<LOD	<LOD	<LOD	<LOD	<LOD	<LOD	<LOD	1.8	<LOD
2000-2003	15.8	2.2	3.7	5.9	5.4	0.9	<LOD	0.2	0.2	<LOD	<LOD	<LOD	<LOD	<LOD	1.1	<LOD
2003-2007	37.8	3.1	5.1	8.4	6.0	2.2	<LOD	<LOD	<LOD	<LOD	<LOD	<LOD	<LOD	<LOD	<LOD	<LOD
2007-2009	155.5	2.9	5.0	7.2	8.6	2.9	0.6	0.2	<LOD	<LOD	<LOD	<LOD	<LOD	<LOD	1.2	<LOD
2009-2013	178.4	2.3	2.9	4.5	7.3	4.8	0.6	0.3	<LOD	<LOD	0.1	<LOD	<LOD	<LOD	1.0	<LOD
2013-2013	193.5	5.2	5.7	5.8	8.5	3.5	<LOD	<LOD	<LOD	<LOD	<LOD	<LOD	<LOD	<LOD	3.4	<LOD
2013-2017	269.2	4.7	5.3	8.0	6.3	3.0	<LOD	<LOD	<LOD	<LOD	<LOD	<LOD	<LOD	<LOD	2.0	<LOD
2013-2017	280.6	3.6	4.9	8.0	7.0	3.9	1.3	0.4	0.4	<LOD	0.1	<LOD	<LOD	<LOD	0.7	<LOD

Red values indicate those analytes that were below method detection limits

Figure S6. Spearmans rank correlation of PFAAs in firn samples at Kohnen Station (1958 – 2017)

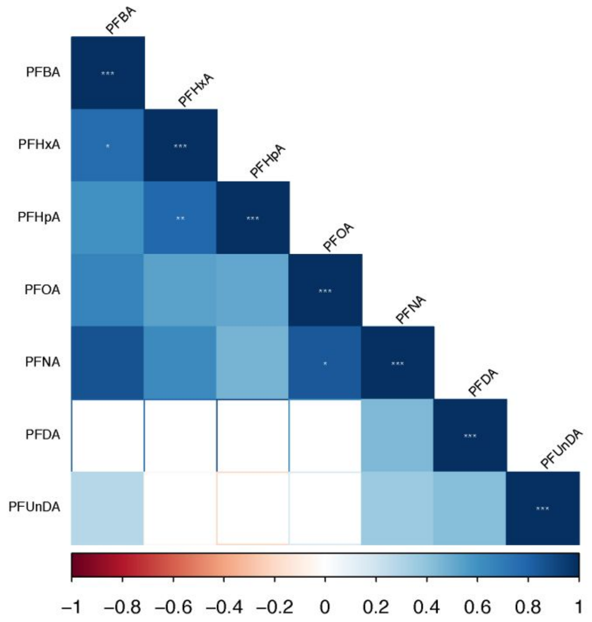

Data analysis was performed on chemical concentration data (pg/L) and only values >MDL were included. Normality was checked using the Shapiro-Wilk test (α = 0.05) and a non-parametric test (Spearmans rank) was applied due to some chemical data not being normal. Only correlations with more than 50% of complete pairwise observations are displayed in correlogram. Asterisk denotes those pairwise observations that are statistically significant (* = p<0.05; ** p<0.01; *** p<0.001).

	C4/C5	C6/C7	C8/C9	C10/C11	C12/C13
1958-1963	1.1	1.0	8.7	-	-
1963-1968	0.9	1.4	5.8	-	-
1968-1975	2.0	1.5	2.5	3.1	4.5
1975-1983	1.0	0.9	3.2	0.4	-
1983-1985	2.2	0.8	7.4	2.4	2.7
1987-1991	0.7	1.3	3.7	-	-
1991-1994	1.4	1.0	5.8	0.8	-
1994-1997	1.0	0.6	6.5	1.5	-
1997-2000	1.2	0.4	4.8	-	-
2000-2003	7.3	0.6	5.8	-	-
2003-2007	12.0	0.6	2.8	-	-
2007-2009	52.9	0.7	3.0	3.0	-
2009-2013	76.1	0.6	1.5	2.3	-
2013-2013	37.1	1.0	2.4	-	-
2013-2017	65.9	0.6	1.9	2.9	-
Mean (± 1.s.d.)	18 ± 27	0.9 ± 0.3	4.4 ± 2.2	2.0 ± 1.0	3.6 ± 1.3

Table S14: Depositional flux ratios for PFCAs homologues.

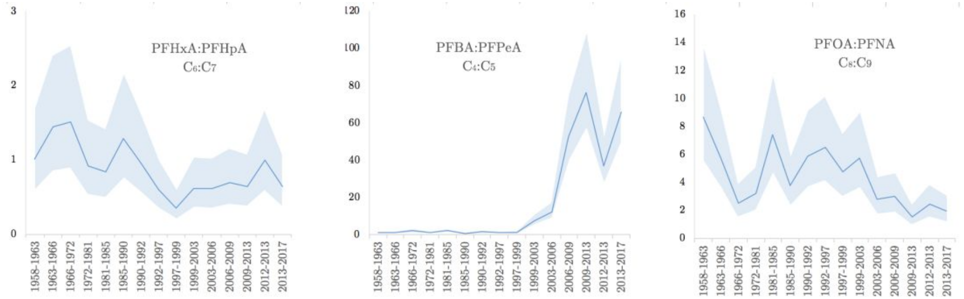

Figure S7: Time-series of depositional flux ratios for even-odd PFCA. Shading indicates combined uncertainty (±1.s.d.) derived from repeatability and reproducibility samples.

Average depositional flux ratios for PFCAs were calculated for the timeseries. Blank values (-) indicate those samples that had at least one homolog less than method detection limit. Depositional flux ratios close to one suggest that FTOHs are a significant source for those particular PFCAs (e.g. C6/C7).

Table S15: Estimated total mass of PFAA deposited in Antarctica between 2013 – 2017

Perfluoroalkyl acid (PFAA)	Annual deposition flux (kg)
PFBA	8892
PFPeA	135
PFHxA	165
PFHpA	259
PFOA	215
PFNA	112
PFDA	41
PFUnDA	14
PFDoDA	12
PFTrDA	<LOD
PFTeDA	4
PFBS	<LOD
PFHxS	<LOD
PFHpS	<LOD
PFOS	<MDL
PFDS	<LOD

Average concentrations in the most recent snow sample (2013 - 2017) and modelled annual turnover of snow in Antarctica of 2100 Gt (SWE; snow water equivalent) (Rignot et al., 2019) were used to estimate the annual deposition flux (kg) of individual PFAAs deposited across the entire continent of Antarctica in a single year. This provided a basis upon which to determine chemical emissions/production rates and thus propose credible chemical sources. These annual deposition fluxes calculated using this simple method are most likely an underestimate of most PFAAs in Antarctica due to the lower concentrations of PFAAs measured in snow at Kohnen (East Antarctic Plateau) compared with coastal regions (Antarctic Peninsula) (Casal et al., 2019, Casal et al., 2017)

References:

CASAL, P., CASAS, G., VILA-COSTA, M., CABRERIZO, A., PIZARRO, M., JIMÉNEZ, B. & DACHS, J. 2019. Snow Amplification of Persistent Organic Pollutants at Coastal Antarctica. *Environmental Science & Technology,* 53**,** 8872-8882.

CASAL, P., ZHANG, Y., MARTIN, J. W., PIZARRO, M., JIMÉNEZ, B. & DACHS, J. 2017. Role of Snow Deposition of Perfluoroalkylated Substances at Coastal Livingston Island (Maritime Antarctica). *Environmental science & technology,* 51**,** 8460.

GÖKTAS, F., FISCHER, H., OERTER, H., WELLER, R., SOMMER, S. & MILLER, H. 2002. A glacio-chemical characterization of the new EPICA deep-drilling site on Amundsenisen, Dronning Maud Land, Antarctica. *Annals of Glaciology,* 35**,** 347-354.

GREMMEL, C., FRÖMEL, T. & KNEPPER, T. P. 2017. HPLC–MS/MS methods for the determination of 52 perfluoroalkyl and polyfluoroalkyl substances in aqueous samples. *Analytical and Bioanalytical Chemistry,* 409**,** 1643-1655.

MUNOZ, G., GIRAUDEL, J. L., BOTTA, F., LESTREMAU, F., DÉVIER, M. H., BUDZINSKI, H. & LABADIE, P. 2015. Spatial distribution and partitioning behavior of selected poly- and perfluoroalkyl substances in freshwater ecosystems: a French nationwide survey. *Sci Total Environ,* 517**,** 48-56.

RIGNOT, E., MOUGINOT, J., SCHEUCHL, B., VAN DEN BROEKE, M., VAN WESSEM, M. J. & MORLIGHEM, M. 2019. Four decades of Antarctic Ice Sheet mass balance from 1979–2017. *Proceedings of the National Academy of Sciences,* 116**,** 1095.
